# Growth, physiology and yield of durum wheat (Triticum durum) treated with sewage sludge under water stress conditions

**DOI:** 10.17179/excli2014-715

**Published:** 2015-02-26

**Authors:** Sonia Boudjabi, Mohammed Kribaa, Haroun Chenchouni

**Affiliations:** 1 Department of Natural and Life Sciences, Faculty of Exact Sciences and Natural and Life Sciences, University of Tebessa, 12002 Tebessa, Algeria; 2 Department of Natural and Life Sciences, Faculty of Sciences, University of El Hadj Lakhdar, 05000 Batna, Algeria; 3 Department of Biology, Faculty of Sciences, University of Setif 1, 19000 Setif, Algeria

**Keywords:** Durum wheat Triticum durum, sewage sludge, chlorophyll a, yield, biomass, water stress

## Abstract

In arid and semi-arid areas, low soil fertility and water deficit considerably limit crop production. The use of sewage sludge as an organic amendment could contribute to the improvement of soil fertility and hence the agronomic production. The study aims to highlight the behaviour of durum wheat to the application of sewage sludge associated with water stress. The assessment focused on morphophysiological parameters of the wheat plant and yield. Under greenhouse conditions, the variety Mohamed Ben Bachir was treated by four water stress levels (100 %, 80 %, 50 % and 30 %). Each stress level comprised five fertilizer treatments: 20, 50 and 100 t/ha of dry sludge, 35 kg/ha of urea, and a control with no fertilization. Results revealed a significant loss in water content and chlorophyll *a* in leaves. Water stress negatively affected the development of wheat plants by reducing significantly seed yield, leaf area and biomass produced. Plant’s responses to water stress manifested by an accumulation of proline and a decrease in total phosphorus. However, the increasing doses of sewage sludge limited the effect of water stress. Our findings showed an increase in the amount of chlorophyll pigments, leaf area, total phosphorus, biomass and yield. In addition, excessive accumulation of proline (1.11 ± 1.03 µg/g DM) was recorded as a result of the high concentration of sludge (100 t/ha DM). The application of sewage sludge is beneficial for the wheat crop, but the high accumulation of proline in plants treated with high dose of sludge suggests to properly consider this fact. The application of sludge should be used with caution in soils where water is limited. Because the combined effect of these two factors could result in a fatal osmotic stress to crop development.

## Introduction

In arid and semi-arid regions of the Mediterranean, water scarcity, combined with fragile skeletal soils and of low organic matter, remains the limiting factor for the development of vegetation in general and cereal crops in particular (Mäder et al., 2002[38]). Indeed, the wheat crop which is very dependent on water lies directly affected from 50 to 90 % (Chennafi, 2012[12]), leading to a steady decline in performance. Agriculture is now seen, not only subject to climatic hazards, but also to poor cultural practices which affect the ability to restore its storage in organic and mineral matters, and water, and thus the biological fertility (Li et al., 2013[34]).

Due to this situation, wheat, overwhelmingly consumed in several countries, including Algeria, faces serious difficulties leading to a deficiency in the production (Jacobsen et al., 2012[24]). Furthermore, the climatic conditions in arid and semi-arid areas are characterized by moderate rainfall, mainly in winter, and a hot dry summer (Bradai et al., 2015[7]). Moreover, the coincidence of maximum heat with the period of rainfall deficit triggers a water stress always marked in these regions (Jacobsen et al., 2012[24]). Because of the low soil moisture, the diffusion of nutrients, even those normally being mobile, can be slowed causing salinization and resulting in removal of vegetation cover under osmotic stress effect and degradation in soil functions (Chennafi et al., 2006[13]).

It is well known that the conciliation of performance improvement of wheat crops depends on maintaining the stock of nutrients in soil, which is essential for plant growth, and the use of conventional resources addressing the problem of water deficit (Huang et al., 2005[22]; Casado et al., 2006[10]; Chennafi et al., 2011[14]). In this regard, several studies have shown that, by their richness in organic matter, sewage sludge helps improve the mineral and water statuses of soil and therefore increases crop production (Lobo et al., 2013[36]). Thus, their long agricultural use is still required at present.

Moreover, the soil is a natural resource whose exploitation should be seen through a conservative wise-use approach limiting all forms of degradation especially in arid and semi-arid areas where fragility and poverty in nutrients are the main soil features (Neffar et al., 2014[41]). This often encourages farmers to undertake a conservation management based on the use of different kinds of waste compounds to improve the performance of their crops, such as cattle or poultry manures and composts (Oustani et al., 2015[47]).

To meet this objective, the incorporation of sludge is an effective alternative which is able to sustainably improve the physical and chemical soil fertility (Courtney and Mullen, 2008[16]; Shaheen et al., 2014[53]). In fact, the application of sewage sludge protects against soil degradation and promotes better plant growth by increasing their potential for survival during drought (Fernández et al., 2007[17]; Van Zwieten et al., 2010[62]). Currently, the majority of studies on sludge (e.g. Bresson et al., 2001[8]; Orman et al., 2014[46]) are oriented towards the agricultural use due to their high intake of nutrients. However, the presence of minerals at high levels generates some understandable concern when it comes to spreading waste on land already subjected to environmental stress (Lassoued et al., 2014[31]).

Within that problematic, the present work aims to study the impact of land application of sewage sludge on the behaviour of durum wheat, through some morphophysiological traits (biomass, relative water content), biochemical parameters (proline and chlorophyll *a*) and yield resulted of the application of increasing doses of sludge associated with a gradient of water deficit.

Thus, we assume that morphophysiological, biochemical parameters and yield will be higher in sludge amended soils compared to control; but also these parameters will improve with increasing sludge amendments, since soil characteristics are known to improve with organic amendments containing sewage sludge (Lobo et al., 2013[36]; Li et al., 2014[35]). The problem posed is whether the plant can supply itself under water stress conditions in a better way with an application of waste sludge than in the absence of fertilization. We expect an increase in the water capacity of soil with the increasing application of sludge, allowing the restoration of water and mineral resources for plants. However, the high mineral filler of that biosolid may establish, at high dose, an osmotic stress effect limiting the development of culture.

## Materials and Methods

### Description of the experimental test

The experiment was conducted in a greenhouse at the Faculty of Exact Sciences and Natural and Life Sciences at the University of Tebessa (Northeastern Algeria). The study lasted five months between February and June 2012. The wheat seed (*Triticum durum* Desf. var. Mohamed Ben Bachir) were recuperated from The Algerian Interprofessional Office of Cereals (OAIC) of Tebessa. The study variety was selected for its resistance to drought and its importance as a staple in the manufacture of semolina, the essential material in the diet of almost Algerian population (Kezih et al., 2014[29]).

The sewage sludge used is activated sludge collected from the wastewater treatment plant of Ain Sfiha (Setif, Northeast Algeria). The soil is removed from the Faculty of Exact Sciences, Nature and Life University of Tebessa. The physicochemical characteristics of the sludge and soil used in experiment were described in an earlier essay by the same authors (Boudjabi and Kribaa, 2012[6]).

In this essay, two factors were considered, water stress and fertilizing treatment. The experimental design consisted in the application of four water stress levels using plastic pots filled with 5 kg of soil. At 5 cm deep of soil, ten wheat seeds were homogeneously sown. The pots were divided into four water regimes: 100 %, 80 %, 50 % and 30 % of field capacity (FC). For each level of water stress, a fertilizer amendment based sludge and urea was applied as follows: 

(i) a sewage sludge treatment, including three levels 56.67, 141.67 and 283.33 g dry matter ‘DM’ of sludge per pot, which respectively are equivalent to 20, 50 and 100 tons of sludge per hectare; 

(ii) a treatment without sludge containing only a supply of mineral fertilizer ‘urea’ with a dose of 0.15 g/pot, which corresponds to 35 kg N/ha; and 

(iii) control without fertilizer amendment.

Each fertilizer treatment was performed four times for each stress level. So that for each level of water stress, a total of 20 pots was processed.

### Collection of the plant material

The first collection of plants took place at the stage of full heading. In each pot, four plants were randomly harvested. Half was used to estimate the leaf area, aboveground dry biomass and relative water content. After drying, these plants have been used for the determination of total phosphorus. The second half of the plants used for the measurement of proline content and the extraction of chlorophyll *a*. The second plant collection occurred at the stage of maturity. The six remaining plants in each pot were retained for yield estimation.

### Morphophysiological parameters

The estimate of the relative water content (RWC) was based on the use of two leaves taken from the two plants collected from each pot. Turgor of cells was determined by measuring fresh weight, and then the leaves were placed in distilled water for 24 hours to have the turgid weight then dried in an oven at 85 °C for 24 hours until obtaining a constant dry weight. The RWC was calculated using the following formula (Barrs, 1968[3]): RWC (%) = (fresh weight – dry weight) (turgid weight – dry weight) × 100.

The two plants that were used for the determination of the RWC were used to calculate the biomass. This variable was assessed for each pot as the average of the dry weights of both dried parts of plants. The weight of leaves taken for RWC was also added for the estimation of dry weight.

We used the method of Paul et al. (1979[48]) for measuring leaf area (LA). The technique consisted in placing the leaf on tracing paper, cut edges of the paper, weigh the portion of the paper representing the leaf (WL). Then we determined the weight WP corresponding to a surface SQ known to a square of the same tracing paper. We deduced leaf area as: LA = (WL–SQ)/WP.

### Determination of biochemical parameters

The method used to calculate the content of proline in leaves is that of Troll and Lindsley (1955[59]) simplified by Wittmer (1987[64]). A 100 mg sample of fresh material was placed in test tubes containing 2 ml of methanol (40 %). The tubes were placed in a water bath at 80 °C for one hour. After cooling, 1 ml taken from the resulting solution was added to 1 ml of acetic acid and 1 ml of the mixture containing 120 ml distilled water + 300 ml of ortho-phosphoric acid. Tubes were placed in the water bath until boiling for 30 min. Once cooled, a volume of 5 ml of toluene was added in. Two phases were separated, the upper organic phase which contained proline was recovered. The absorbance of the letter was measured on an UV–VIS–1205 spectrophotometer at a wavelength of 528 nm. The concentration of proline was derived from the calibration curve: y = 0.91x + 0.0043, R² = 0.989.

For the calculation of the chlorophyll *a*, a 100 mg sample of fresh material obtained from leaves was ground in the presence of acetone (80 %). After filtration, the optical density (OD) is measured at 663 and 665 nm. The concentration of chlorophyll *a* was then derived as follows: Chlorophyll *a* (mg/kg DM) = 12 (OD_663_) – 2.67 (OD_645_) (Wittmer, 1987[64]).

The total phosphorus was determined using the method of Olsen et al. (1954[45]). In a muffle furnace, 0.5 g of milled plants were calcined for two hours at 500 °C. After an acid attack of the sample with concentrated HCl and a wash with distilled water, a stock solution was obtained (100 ml). For each sample of the stock solution 1.5 ml were taken, to which was added 6.5 ml of ascorbic acid, 2 ml of sulphomolybdate and 1 ml of distilled water. After incubation, an absorbance reading was performed on a spectrophotometer at 650 nm. The concentrations were deducted from the calibration curve: y = 0.032*x* + 0.1709, *R²* = 0.873.

### Seed yield

The six plants obtained at the end of the experiment were used to calculate the seed yield (SY) using the following formula: SY = number of seeds per spike × average seed weight.

### Statistical analysis

The data obtained from the experiment for each wheat variable were represented by the mean with standard deviation (SD) following levels of water stress and fertilizer treatments. Two-way ANOVAs were applied to test the effects of the two factors ‘water stress’ and ‘fertilizer treatment’ and their interaction ‘stress × treatment’ on the variation of the seven morphophysiological and biochemical variables measured. The Tukey’s post hoc test was carried out to classify levels of factors with a significant effect (*P* < 0.05). In addition, to test the relationship between the various parameters of wheat, Pearson correlation tests were applied between these parameters in pairs. To test the relationship between the various parameters of wheat, two sided Pearson correlation tests were applied between these parameters in pairs. Statistical analyses were performed with the help of the R software (R Core Team, 2014[50]) using the functions ‘aov’ for ANOVA, ‘TukeyHSD’ for the Tukey's post hoc test, and ‘rcorr.adjust’ to compute the correlation matrix of Pearson.

## Results

### Aboveground biomass

The analysis of variance showed that the water deficit (F_(3,60) _= 13.64, *P* < 0.001), fertilizer treatment (F_(4,60)_ = 47.68, *P *< 0.001) and their interaction (F_(12,60)_ = 4.66, *P* < 0.001) were well expressed by their highly significant effects on the variation of aboveground biomass values.

The Tukey HSD test applied for averages of biomass between water stress levels revealed the following three groups in descending order: FC1 > FC2, FC3 > FC4. Plant biomass observed in the control pots (1.05 ± 0.70 g DM/plant) was significantly greater than averages of the other levels of irrigation FC2, FC3 and FC4 that formed a homogeneous group with 0.85 ± 0.52, 0.68 ± 0.28 and 0.30 ± 0.60 g DM/plant, respectively (Table 1). Biomass values recorded at FC2 and FC3 were intermediate between FC1 and FC4.

For fertilizers, mainly sewage sludge ‘SS’ applied at 50 and 100 t/ha that showed a significant improvement in terms of biomass. The multiple comparisons of biomass means between SS levels revealed the highest values (1.46 ± 0.71 g DM/plant) with SS3, followed by SS2 level with 0.95 ± 0.26 g DM/plant, which both were significantly higher than mean biomasses recorded at SS1 level, urea and control plants (Table 1(Tab. 1)).

### Leaf area

The analysis of variance of leaf area results between water stress levels (F_(3,60)_ = 79.38, *P* < 0.001), fertilizer treatments (F_(4,60)_ = 279.51,* P* < 0.001) and their interaction ‘stress × treatment’ (F(_12,60) _= 15.03, *P* < 0.001) were revealed highly significant. Tukey’s test identified two groups of leaf area means between water stresses. The first level ‘FC1’ significantly denoted the highest value of leaf area (11.69 ± 9.87 cm²) and the FC4 the lowest value (4.40 ± 3.66 cm²), whereas values of FC2 and FC3 levels were intermediary between previous levels. 

The increased application of sewage sludge promoted the increase of leaf area unlike the mineral fertilizer that showed no significant difference compared to control and level SS1, which all were included as a homogeneous group following Tukey test: control, urea, SS1 < SS2 < SS3. Indeed, leaf area gradually increased with the increase of SS doses, starting from 4.83 ± 1.72 cm² at SS1, then 10.17 ± 4.61 cm² at SS2 to reach up 18.52 ± 7.59 cm² at SS3, but at the same time, it decreased following the increase of water stress (Table 2(Tab. 2)).

### Relative water content (RWC)

The RWC in leaves decreased along the increase of water stress but increased with the increase of SS doses. Overall, the RWC in wheat leaves of our experiment was 50.89 ± 17.29 %. The ANOVA revealed a significant difference of RWC between water stress levels (F_(3,60)_ = 21.94, *P* < 0.001), whereas Tukey test revealed three homogeneous groups of them: FC1 level gave the highest values with a mean 66.90 ± 15.61 %, followed by FC2 with 51.22 ± 11.2 %, then came FC4 with the lowest value (37.69 ± 13.43 %). RWC at FC3 level was 47.75 ± 15.16 %, it was placed intermediary between the two last levels of stress (Table 3(Tab. 3)). 

The effect of fertilizer treatments was also significant on variation of RWC of leaves (F_(4,60)_ = 9.72, *P* < 0.001). The RWC was significantly higher in plants treated with SS ‘group C’ compared to urea and control ‘group A’. The highest value of RWC was noted at SS3 dose with 59.75 ± 19.31 %, whereas the lowest recorded at control with 40.33 ± 14.75 %, the treatment SS2, SS1 and urea represent group B following Tukey test, and revealed average RWC values compared to SS3 and control (Table 3(Tab. 3)). The effect of the interaction ‘stress × treatment’ was statistically not significant on variation of RWC (F_(12,60) _= 0.98, *P* = 0.479).

### Proline

The accumulation of proline in leaves increased with the increase of water stress levels and also the increase of doses of SS compared to control and urea, where the highest value (2.77 ± 0.48 µg/g DM) was recorded in plants treated with FC4 and SS3. Means of proline showed significant differences between levels of water stress (F_(3,60)_ = 294.25; *P *< 0.001) and between fertilizer treatments (F_(4,60)_ = 4.49, *P* = 0.003). However, the ANOVA revealed that variations of proline values were not significant for the interaction ‘stress × treatment’ (F_(12,60) _= 1.56, *P* = 0.129). Tukey test showed that values for this osmoticum in the leaves were higher under FC4 (2.47 ± 0.57 µg/g DM), then FC3 (0.65 ± 0.17 µg/g DM) then both FC2 and FC1 (0.31 ± 0.13 and 0.20 ± 0.12 µg/g DM, respectively) that were significantly not different. Regarding fertilizer treatment, the level SS3 produced the highest proline amount in leaves (1.11 ± 1.03 µg/g DM), followed by SS2 with 1.00 ± 1.11 µg/g DM. Concentrations of proline in plants treated with the two previous levels were significantly greater than those treated with SS1, control and urea, respectively, in which the proline were 0.89 ± 1.00, 0.79 ± 1.01 and 0.76 ± 0.78 µg/g DM, respectively (Table 4(Tab. 4)).

### Chlorophyll a

ANOVAs revealed a significant variation of chlorophyll *a* between levels of water stress (F_(3,60)_ = 18.55, *P* < 0.001) and between fertilizer treatments (F_(4,60)_ = 29.44, *P* < 0.001). Overall, the chlorophyll *a* content of the study variety was 1.87 ± 0.66 mg/kg of DM. The plants irrigated with 100 % and 80 % field capacity showed the highest concentrations of chlorophyll *a* with 2.20 ± 0.59 and 2.06 ± 0.44 mg/kg DM, respectively. These values decreased gradually as the water stress increased to reach the lowest value of 1.38 ± 0.77 mg/kg DM at 30 % of FC. FC3 revealed 1.84 ± 0.52 mg of chlorophyll *a* per kg of plant DM; following Tukey test, the value recorded at that level was classified between FC1–FC2 and FC4 (Table 5(Tab. 5)). Moreover, the effect of the interaction of the two studied factors was not significant (F_(12,60)_ = 1.25, *P* = 0.274). The effect of fertilizer amendment significantly increased the concentration of chlorophyll *a* in wheat leaves. The application of SS with 50 and 100 t/ha significantly induced a higher production of chlorophyll *a* compared with plants treated with SS1, urea and control, in which values were significantly not different in Tukey's test (Table 5(Tab. 5)).

### Total phosphorus

The effect of water stress was significant (F_(4,60) _= 10.01, *P* < 0.001) on variation of total phosphorus contents of leaves. This parameter decreased while water stress increases. Tukey's test classified means of phosphorus into two groups of stress levels; FC1 denoted the highest value (0.242 ± 0.009 %) and FC4 the lowest value (0.237 ± 0.003 %), FC2 and FC3 were intermediary between those two. As for fertilizer treatments, the ANOVA indicated a highly significant effect (F_(4,60)_ = 42.5, *P* < 0.001). Multiple comparisons of phosphorus means revealed a significant increase of values, particularly when SS was applied compared to urea and control which were significantly not different and both denoted the lowest values (0.235 ± 0.001 %). In addition, Tukey’s test specified that total phosphorus was higher in plants treated with SS3 (0.248 ± 0.010 %) compared to those of SS2 and SS1 where phosphorus was 0.241 ± 0.003 % and 0.238 ± 0.001 %, respectively (Table 6(Tab. 6)). The effect of the interaction of water stress and fertilizer treatment was significant (F_(12,60)_ = 3.8,* P* < 0.001) on total phosphorus content of leaves.

### Seed yield

Values of seed yield significantly differed between levels of water stress (F_(3,60) _= 51.21, *P *< 0.001), fertilizer treatments (F_(4,60)_ = 42.52, *P* < 0.001) and the interaction of both study factors (F_(12,60)_ = 5.61, *P *< 0.001). The studied variety generally yielded 1.10 ± 1.08 g/plant, on average. Regarding water stress levels, the Tukey’s test identified two groups of stress levels. The mean of yield in FC1 (1.95 ± 1.43 g/plant) was significantly higher than FC2 and FC3 (1.45 ± 0.94 and 0.60 ± 0.39, respectively), which were also higher than FC4 (yield = 0.41 ± 0.36 g/plant). The application of SS significantly increased seed yield, but starting from the treatment SS2 (50 t/ha) onward. Values of yield recorded in plants treated with SS3 (2.04 ± 1.29 g/plant) and then SS2 (1.74 ± 1.31 g/plant) were statistically higher than those of SS1, urea and control, which were all three not different following Tukey’s test (Table 7(Tab. 7))

### Relationships between morphophysiological traits of wheat

The correlations between all morphophysiological parameters of durum wheat were positive and highly significant (*P* < 0.001), except with proline where all values of Pearson coefficient were negative and only significant with WRC, chlorophyll *a* and seed yield (Table 8(Tab. 8)). 

## Discussion

Under water deficit, the depressive effect of the biomass produced by plants indicates that they adjust their sizes according to the amount of water available in the habitat. This is a major trait adapted by plants to reduce the need for water when the latter is insufficient (Ferryra et al., 2004[18]; Lebon et al., 2006[32]; Locke and Ort, 2014[37]). The significant positive correlation between leaf area trained and aboveground biomass indicates that the plants in order to reduce their water requirements adopt a reduction in the evaporative surface of their leaves (Bouchabke et al., 2006[5]). In this sense, several studies reported that the reduction of the leaf surface under water stress may be due to a decrease in the mitotic activity of epidermal cells which result in a reduction in total number of leaf cells (Chartzoulakisa et al., 2002[11]; Saab and Sharp, 2004[52]; Locke and Ort, 2014[37]).

It is clear that the amendment of sludge increased the production of dry matter and leaf surface in durum wheat. This may be explained by the fact that this biosolid has great potential in improving the nutritional quality of soil (Nielson et al., 1998[42]; Singh and Agrawal, 2007[56]; Van Zwieten et al., 2010[62]). The sewage sludge is rich in nutrients that plants need for their development, particularly the anions and cations (Sing and Sinha, 2002[55]). The SS2 and SS3 levels (50 and 100 t/ha) were more efficient and provided high and significant improvement in terms of biomass, this is due to the high availability of nutrients which is based on sludge doses (Levi-Minzi et al., 1999[33]; Lobo et al., 2013[36]). Our findings corroborate those of Monreal et al. (2007[40]), which reported the positive contribution of sewage sludge on the production of dry matter and leaf area of plants. It also shows that the mineral fertilizer ‘urea’ was not valued as well in the development of leaves than the aboveground biomass. The lack of water in the soil has a decisive effect that induces a decrease in its content in plants (Huang et al., 2005[22]). This decrease is due to the dehydration phenomenon that affects cells (Brossa et al., 2013[9]).

In addition, our results indicates that the water reserves for plants that match the level FC2 (80 %) and FC3 (50 %) are similar and therefore are classified in the same group. This is explained by the fact that when soil water content is higher than 30–40 %, plant transpiration is little affected (Mata-Gonzalez et al., 2002[39]). Our findings confirm those of Iannucci et al. (2000[23]) reporting under water deficit cell turgor for a forage plant (*Trifolium alexandrinum L.*) equal to 68 % of the control plants and 60 % for stressed plants. Through sludge-treatment improved soil moisture unlike the control pots and those treated with mineral fertilizer, water restoration for plants amended by the biosolid was higher, and that all three doses of sludge improve the water content of cells and maintain a higher turgor than the control and that of urea. Similarly, several studies confirm that the organic matter of sewage sludge contributes in the improvement of physical properties of the soil by increasing its water-holding capacity (Singh and Agrawal, 2007[56]; Fiasconaro et al., 2013[19]).

In our essay, the sludge was applied on the surface of soil, this type of application create a real mulch that limits the effect of evaporation and helps to retain moisture longer and consequently to maintain a higher level of turgor in sludge-treated plants compared to control plants and fertilized with urea (Splawski et al., 2014[58]).

Furthermore, the adjustment of leaf area, biomass and proline accumulation represent a real defence mechanism adopted by plants to cope with water stress of which they are subjected (Gregory et al., 2000[21]; Planchet et al., 2014[49]). Under water stress, the relative water content of plants gradually decreases because of the difficulties they face in order to restore this important source. In our study, the correlation obtained between the RWC and produced proline (*r* = –0.432,* P* < 0.001) reflects that plants synthesize this amino acid in order to tolerate the lack of water (Monreal et al., 2007[40]; Kakati et al., 2013[26]; Planchet et al., 2014[49]). Our results are similar to those obtained by Kazama et al. (2014[28]) studying *Arabidopsis thaliana* and Ullah et al. (2014[61]) investigating wheat. Proline is an osmotic adjustment mediator that allows plants to stabilize cellular structures (Brossa et al., 2013[9]). The production of this amino acid has been demonstrated in many species and in different situations of osmotic, water and heat stresses (e.g. Ain-Lhout et al., 2001[1]; Raymond and Smirnoff, 2002[51]; Planchet et al., 2014[49]).

The effect of fertilizer treatment on proline synthesis in leaves showed that the application of sludge provides significant accumulation compared to the control. These results are explained by the fact that the sludge is considered as a high source of nitrogen (Lobo et al., 2013[36]), a basic element in the formation of proline. In this context, our results are similar to those of Antolin et al. (2005[2]) which indicated an increase in the proportion of plant proteins when the sewage sludge was applied. The Tukey’s test revealed that the SS3 level (100 t/ha) shows a higher accumulation of proline in leaves unlike other fertilizer treatments. This effect is obviously related to the high doses of minerals contained in this level of treatment, which, by this intake created in addition to the water deficit, an osmotic stress that encouraged an excess in the accumulation of proline (Çiçek and Çakirlar, 2002[15]; Kakati et al., 2013[26]).

Alongside to the accumulation of proline, the inverse proportionality found between this amino acid and chlorophyll *a* (correlation: *r *= –0.351, *P* = 0.001) suggests the existence of a plausible connection between the biosynthetic pathways of these two compounds that is summarized by a competition for the glutamate (Bengson et al., 1978[4]). Therefore, the more proline is accumulated the more there is a sharp decrease in chlorophyll pigment. In our study, the water deficit implies a decrease in chlorophyll* a* contents due to the diminution of the opening of leaf stomata to limit the effect of water loss through transpiration phenomenon. This effect leads to a dilution of chlorophyll (Kasraoui et al., 2006[27]; Locke and Ort, 2014[37]). Indeed, under severe water stress, the transport of oxygen electrons and decrease in photochemical quenching are unable to dissipate the excess of excitation energy, thus causing photo-damages at the level of PSII (photosystem that includes chlorophyll *a*) (Nogués and Baker, 2000[44]).

As for the effect fertilizing treatments, sludge provides an improvement in chlorophyll *a* which is more important at high sludge doses, because simply proper nutrition induces good photosynthetic activity (Mata-González et al., 2002[39]; Jannoura et al., 2014[25]). It is noteworthy mentioning that during our experiment, the sludge-treated plants were greener compared with control plants and those treated with urea. Actually, the sludge is a source rich of several essential elements entering in the composition of the chlorophyll, such as zinc, iron and magnesium (Korboulewsky, 2002[30]; Orman et al., 2014[46]). Iron is present mainly in young leaves where it is involved in the formation of chlorophyll, while manganese is necessary for normal plant development because it is linked to iron in its action related to the formation of chlorophyll. Both are base molecules involved in the formation of the pyrrole ring. Regarding the content of phosphorus in plants, it is obvious that the effect of water deficit limits its availability to plants. Water is a solvent that allows dissolution of minerals in the soil. Therefore its reduction limits its role and makes the absorption of minerals by plants very hard (Chennafi et al., 2006[13]; Fini et al., 2013[20]).

Indicators of phosphorus bioavailability are the change in biomass production and/or the variation in total amount of phosphorus taken up by the crop. In our study, the increase in the produced biomass suggests a good availability of the mineral that is sludge-originated. Moreover, this is seen through the positive correlation (*r* = 0.691, *P* < 0.001) between the aboveground dry matter produced in all pots and phosphorus content, which confirms the idea of the incorporation of this nutrient in the aboveground biomass produced (Sommers and Sotton, 1980[57]). The sewage sludge used in this study are high in phosphorus elements (Boudjabi and Kribaa, 2012[6]), this mineral mainly originates from detergents (Sommers and Sotton, 1980[57]). Thus, once dissolved in soil, it is placed directly on the availability of durum wheat. This dissolution depends on sludge doses applied. According to Xie et al. (2014[65]) phosphorus of sludge has a bioavailability similar to that of soluble inorganic phosphorus; its average fertilizer value is about 87 %. Moreover, our results concerning the accumulation of total phosphorus in plant tissues are different to those of Warman and Termeer (2005[63]), which compared the effect of application of mineral fertilizer and sewage sludge on grass forage and found a dry matter intake of 25 % and 8 %, respectively.

The decrease in seed yield caused by water deficit is explained by the combined action of water stress and fertilizer amendment. Sewage sludge impose into the soil an osmotic stress that induces a deficit in mineral nutrition (of nitrogen and phosphate), mainly by decreasing movement of elements to roots (Chennafi, 2012[14]; Kakati, 2013[26]). The result is a reduction in biomass and the assimilating surface of leaves, subsequently a decrease in yield (Fini et al., 2013[20]).

The improvement of wheat yield when plants threated with high doses of sludge resulted of the absorption of macro- and micronutrients, which are abundantly provided by the sludge (Tsakou et al., 2002[60]; Nogueira et al., 2013[43]). The incorporation of sludge has also a positive effect on the biological and enzymatic activity of the soil (Lobo et al., 2013[36]) and consequently on phosphorus and nitrogen mineralization (Simek, 2000[54]; Casado et al., 2006[10]). Thus, these benefits to the soil allow a gradual increase of the yield of drylands where environmental conditions are severe and soils are naturally poor. In this respect, the spreading of sludge undeniably fertilizes soils and therefore improves the yield of plants (Warman and Termeer, 2005[63]). This may be used as a plan to improve cereal crop production in arid and semi-arid regions, particularly under seasonal or permanent water stress conditions as the case of the rainfed agriculture (Antolin et al., 2005[2]; Chennafi, 2012[14]; Jacobsen et al., 2012[24]).

## Conclusion

In light of our findings, water stress negatively affects physiological and production parameters of wheat plants. This is evident through the accumulation of proline, decrease in leaf area and RWC, and more a dissolution in the pigment of chlorophyll *a*, which induced a loss in biomass. The lack of water limits the content of phosphorus in the dry matter of the plant, which led to a decrease in seed yield.

Sewage sludge reduce the effect of water stress on crops by increasing retention of water content and chlorophyll *a* and also the improvement of aboveground biomass produced by plants. The application of sludge augments the total phosphorus in leaves and then seed yield. However, high dose of sludge induces a stressful osmotic effect due to the hydrophilic effect of the organic matter contained in the sludge. This effect resulted in an excess in proline accumulation to cope this stress. Thus, the application of sewage sludge should be sustainably applied based on the information considering all the technical aspects of its characterization and uses. Indeed, there is no question of spreading the sludge anarchically and in any conditions.

## Notes

Sonia Boudjabi and Haroun Chenchouni (Tel.: +213-779-462-990, Fax: +213-37-497-502, E-mail: ) contributed both as corresponding authors.

## Conflict of interest

The authors declare that they have no conflict of interest.

## Figures and Tables

**Table 1:**
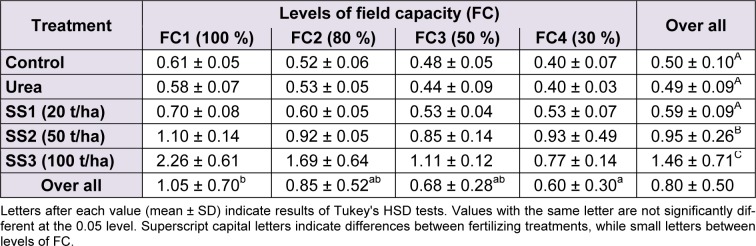
Effect of different levels of fertilizing treatment (control, urea, and 20, 50 and 100 t/ha of sewage sludge ‘SS’) on aboveground biomass (g DM/plant) of durum wheat, under different water stress conditions (100 %, 80 %, 50 % and 30 % of field capacity ‘FC’)

**Table 2:**
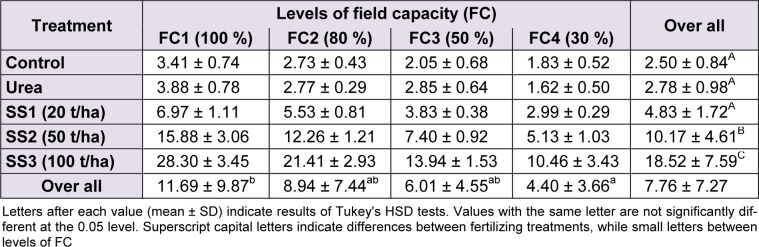
Effect of different levels of fertilizing treatment (control, urea, and 20, 50 and 100 t/ha of sewage sludge ‘SS’) on leaf area (cm²) of durum wheat, under different water stress conditions (100 %, 80 %, 50 % and 30 % of field capacity ‘FC’)

**Table 3:**
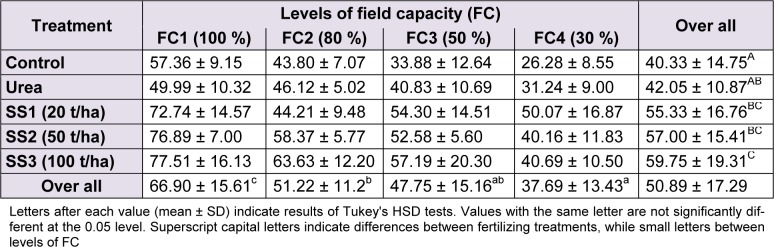
Effect of different levels of fertilizing treatment (control, urea, and 20, 50 and 100 t/ha of sewage sludge ‘SS’) on leaf relative water content (%) of durum wheat, under different water stress conditions (100 %, 80 %, 50 % and 30 % of field capacity ‘FC’)

**Table 4:**
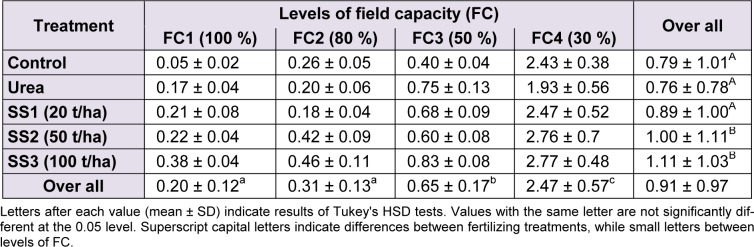
Effect of different levels of fertilizing treatment (control, urea, and 20, 50 and 100 t/ha of sewage sludge ‘SS’) on proline content (µg/g DM) of durum wheat, under different water stress conditions (100 %, 80 %, 50 % and 30 % of field capacity ‘FC’)

**Table 5:**
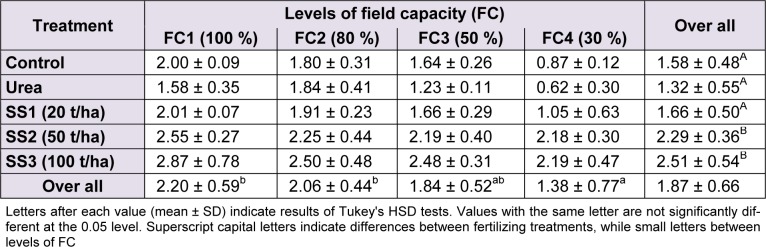
Effect of different levels of fertilizing treatment (control, urea, and 20, 50 and 100 t/ha of sewage sludge ‘SS’) on chlorophyll *a* content (mg/kg DM) of durum wheat, under different water stress conditions (100 %, 80 %, 50 % and 30 % of field capacity ‘FC’)

**Table 6:**
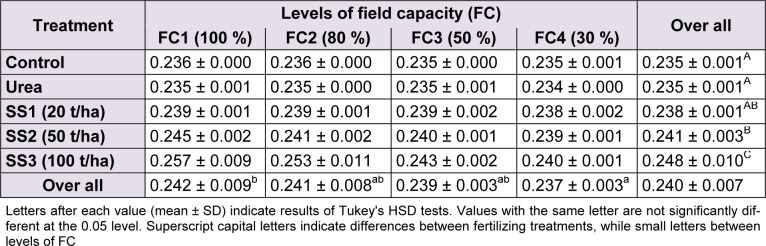
Effect of different levels of fertilizing treatment (control, urea, and 20, 50 and 100 t/ha of sewage sludge ‘SS’) on total phosphorus content (%) of durum wheat, under different water stress conditions (100 %, 80 %, 50 % and 30 % of field capacity ‘FC’)

**Table 7:**
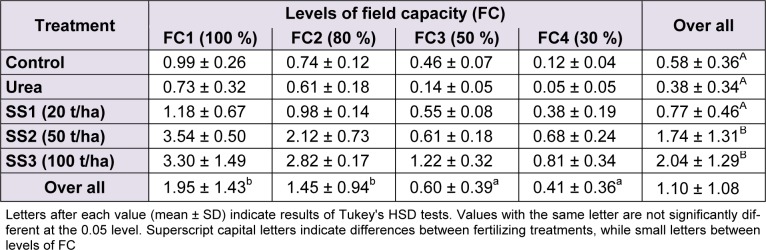
Effect of different levels of fertilizing treatment (control, urea, and 20, 50 and 100 t/ha of sewage sludge ‘SS’) on seed yield (g/plant) of durum wheat, under different water stress conditions (100 %, 80 %, 50 % and 30 % of field capacity ‘FC’)

**Table 8:**
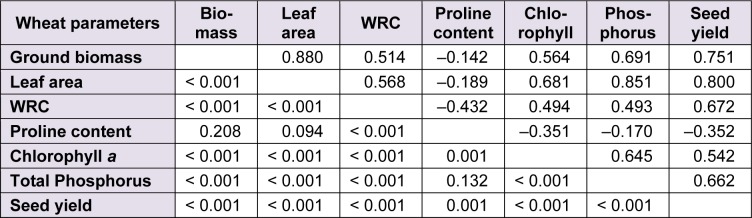
Matrix of Pearson correlations between morphophysiological parameters of durum wheat. The values are referred to Pearson’s correlation coefficient ‘*r*’ (above the diagonal) and the corresponding pairwise two-sided* P*-value (under the diagonal)
